# SARS-CoV-2 Subunit Virus-like Vaccine Demonstrates High Safety Profile and Protective Efficacy: Preclinical Study

**DOI:** 10.3390/vaccines10081290

**Published:** 2022-08-10

**Authors:** Anna V. Vakhrusheva, Aleksandr V. Kudriavtsev, Nickolay A. Kryuchkov, Roman V. Deev, Maria E. Frolova, Konstantin A. Blagodatskikh, Milana Djonovic, Andrey A. Nedorubov, Elena Odintsova, Aleksandr V. Ivanov, Ekaterina A. Romanovskaya-Romanko, Marina A. Stukova, Artur A. Isaev, Igor V. Krasilnikov

**Affiliations:** 1 Betuvax LLC, 121096 Moscow, Russia; 2 Department of Bioengineering, Biological Faculty, Lomonosov Moscow State University, 119991 Moscow, Russia; 3 Clinical Excellence Group LLC, 127051 Moscow, Russia; 4 Department of Pathological Anatomy, I.I. Mechnikov North-Western State Medical University, 195067 Saint Petersburg, Russia; 5 PJSC Human Stem Cells Institute, 129110 Moscow, Russia; 6 Center of Genetics and Reproductive Medicine “Genetico”, 119333 Moscow, Russia; 7 Center for Preclinical Studies, Sechenov First Moscow State Medical University, 119435 Moscow, Russia; 8 Biotechnology Developments LLC, 119285 Moscow, Russia; 9 Department of Vaccinology, Smorodintsev Research Institute of Influenza, Ministry of Health of the Russian Federation, 197376 Saint Petersburg, Russia

**Keywords:** SARS-CoV-2, RBD-based vaccine, nanoparticle vaccine, recombinant vaccine, betulin, COVID-19

## Abstract

Public health threat coming from a rapidly developing COVID-19 pandemic calls for developing safe and effective vaccines with innovative designs. This paper presents preclinical trial results of “Betuvax-CoV-2”, a vaccine developed as a subunit vaccine containing a recombinant RBD-Fc fusion protein and betulin-based spherical virus-like nanoparticles as an adjuvant (“Betuspheres”). The study aimed to demonstrate vaccine safety in mice, rats, and Chinchilla rabbits through acute, subchronic, and reproductive toxicity studies. Along with safety, the vaccine demonstrated protective efficacy through SARS-CoV-2-neutralizing antibody production in mice, rats, hamsters, rabbits, and primates (rhesus macaque), and lung damage and infection protection in hamsters and rhesus macaque model. Eventually, “Betuvax-CoV-2” was proved to confer superior efficacy and protection against the SARS-CoV-2 in preclinical studies. Based on the above results, the vaccine was enabled to enter clinical trials that are currently underway.

## 1. Introduction

The world population is currently struggling with an outbreak of COVID-19 caused by the severe acute respiratory syndrome-related coronavirus 2 (SARS-CoV-2) [1]. The World Health Organization (WHO) reported over 400 million confirmed cases of COVID-19 and up to 6 million deaths by 8 February 2022 [2]. As a result, much effort was made to develop safe and efficient vaccines. By 7 February 2022, there were 183 vaccine candidates developed, and only 10 of them were approved for use by the WHO [3]. Nevertheless, by mid-February 2022, only 61.9% of the world population received at least one dose of a vaccine against COVID-19, that being approximately 10 billion administered doses, but mostly in high-income countries, whereas in low-income countries, only 10.6% [4]. Although, in the past, the only known coronavirus infections were associated with mild symptoms as in a “common cold”, the severe clinical conditions that SARS-CoV-2 causes and the magnitude of the coronavirus pandemic sparked the scientific interest in developing vaccines and antiviral treatments despite the previous scarce research in the field. Moreover, the fast evolution of the SARS-CoV-2 and the emergence of new highly contagious strains like Omicron reclaimed the urgent need to develop safe, effective, and easily scalable vaccines, including those for booster vaccination. 

The SARS-CoV-2 Spike (S) protein is a major component that is required for receptor binding, membrane fusion, and viral penetration. The S protein is processed at the S1/S2 cleavage site by host cell proteases, thus generating the N-terminal S1-ectodomain and a C-terminal S2-membrane-anchored protein. The S1 domain consists of the following subdomains: the NTD (N-terminal domain), the RBD (Receptor-Binding-Domain; residues 319-527), and the S1 and S2 subdomains (SD1, SD2) [5]. The RBD is responsible for the interaction with angiotensin-converting enzyme 2 (ACE2), the primary SARS-CoV-2 receptor that is expressed in many tissues, including type II alveolar epithelial cells [6]. Therefore, the RBD is an antigen eliciting neutralizing antibodies and a major target in vaccine development. 

Currently, there are 40 approved vaccines and around two hundred vaccine candidates [3]. All so-far approved coronavirus vaccines can be classified into one of the following groups: inactivated coronavirus vaccines, non-replicating virus vector-based vaccines, mRNA-based, and protein subunit-based vaccines. 

Inactivated vaccines are produced by the cultivation of SARS-CoV-2 in cell culture and its subsequent inactivation. Their production requires a production line with a very high biosafety level. Such vaccines induce a wide range of cellular and humoral responses due to different viral epitopes that are recognized by immune cells instead of inducing a specific immune response against the pathogen. In addition, for this vaccine type, lower neutralizing antibodies were observed in comparison with the mRNA vaccines [7]. Inactivated vaccines against SARS-CoV-2 are being developed in Russia (CoviVac), China (BBIBP-CorV, CoronaVac), and other countries. The CoronaVac, developed by the Chinese company Sinovac (Beijing, People’s Republic of China), was one of the first vaccines to be approved [8]. 

On the other hand, the non-replicating viral vector-based vaccines, such as, for example, those based on the adenovirus, have good efficacy profiles and relatively persistent immune responses [9]. Despite the high cell tropism and a strong immune response that these vaccines can induce, the genomic integration of viral nucleic acid is highly likely. Therefore, their long-term safety, powerful efficacy, and the possibilities of scaling up the production remain questionable. 

The pre-existing immunity against the viral vaccine vector may impair the vaccine’s immunogenicity and efficacy. Therefore, repeated use or booster vaccination makes this even more complicated. These vaccines may not be as effective for people with recessive infectious viruses [10]. The approved vaccines which pertain to this class are the following: Ad26.COV2.S (Janssen, Beerse, Belgium), Sputnik V and Sputnik Light (Gamaleya National Center of Epidemiology and Microbiology, Moscow, Russia), Vaxzevria (AstraZeneca/Oxford University), Convidecia (CanSino, Tianjin, China), and others. 

RNA vaccines are based on a promising and simple but novel and insufficiently explored platform with several technological barriers. The main concern is related to the stability of the mRNA during vaccine production, storage, and transportation. Due to that, such vaccines require supply chains that conform to the strict temperature requirements [9]. Despite the concern that this technology may lead to genome integration of the administered nucleic acid, this was considered unlikely since there is no interaction between the mRNA and the genomic DNA of the host cell. Nevertheless, some experts find that regulatory assessment of the mRNA-based vaccines should treat these vaccines as gene therapeutics and evaluate them on genotoxicity as well [11]. Other potential drawbacks of the mRNA vaccines include the risk of activation of the RNA-induced interferon response leading to the decline in the vaccine efficacy due to the delay in the mRNA translation and its degradation [12]. In addition, these vaccines have a relatively high proportion of side effects in comparison with other types [10,13]. The main representatives of the mRNA vaccines are mRNA-1273/Spikevax (Moderna, Cambridge, MA, USA) and BNT162b2/Comirnaty (Pfizer/Biontech, Mainz, Germany), which represent the first mRNA-based vaccines ever approved. 

Coronavirus vaccines based on the non-replicating viral vectors and mRNA so far demonstrated the highest efficacies. Nevertheless, these technologies bear a potential risk of genotoxicity and mutagenicity. Some studies indeed demonstrated that host cells could transcribe RNA to DNA, thus enabling genome integration. In addition, there is still no data on the possible long-term adverse effects of any of these COVID-19 vaccines that are currently in use. Meanwhile, the technology of subunit protein-based vaccines is a well-studied platform with a wide number of approved vaccines, including influenza, hepatitis B and C, and papillomavirus [14]. These types of vaccines have a predictable safety profile and good stability [10]. Nevertheless, they often require an additional adjuvant to boost the immune response [14]. An example of this type of COVID-19 vaccine is Nuvaxovid/NVX-CoV2373 (Novavax, Gaithersburg, USA).

That said, we decided to develop a subunit protein-based class of vaccines, “Betuvax-CoV-2”, as the safest one and use VLP-mimicking technology to induce vaccine immunogenicity. The “Betuvax-CoV-2” vaccine is based on RBD and SD1 domains of the S1 protein that are fused with the Fc-fragment of the human IgG1. This vaccine antigen is absorbed on the surface of the 100–180 nm betulin-based spherical particles acting as a vaccine adjuvant and mimicking the SARS-CoV-2 particles [15]. The present paper aims to summarize all preclinical findings pertaining to the “Betuvax-CoV-2” vaccine, i.e., its safety, immunogenicity, and protectiveness. 

## 2. Materials and Methods

### 2.1. Animals & Ethics Statement

BALB/c and C57BL/6 mice that were used in the present research were obtained from the Stolbovaya branch of the Scientific Center of Biomedical Technologies of the Federal Medical and Biological Agency of the Russian Federation. One part of the study was conducted at the Smorodintsev Research Institute of Influenza of the Ministry of Health of the Russian Federation, Saint Petersburg. The experiments on the outbred mice, rats, and rabbits were carried out at the Federal State Autonomous Educational Institution of Higher Education I. M. Sechenov, the First Moscow State Medical University of the Ministry of Health of the Russian Federation, Moscow, Russia. Golden hamsters used in the immunogenicity and safety studies were obtained from the Branch of the Shemyakin–Ovchinnikov Institute of Bioorganic Chemistry of the Russian Academy of Sciences (Nursery for the Laboratory Animals, Puschino, Russia). The experiments on rhesus monkeys were conducted at the Research Institute of Medical Primatology, Sochi, Russia. The animals were infected at the Federal State Budgetary Institution, Central Research Institute No. 48 of the Russian Ministry of Defense, Sergiev Posad, Russia.

The number of animals used in the study was sufficient to assess the safety, immunogenicity, and protective properties of the vaccine, and, at the same time, the number was sufficient to comply with the principles of ethical research. All animals were kept under standard conditions in accordance with the Directive 2010/63/EU of the European Parliament and the Council of the European Union of 22 September 2010, on the protection of animals used for scientific purposes. Animal husbandry was compliant with each facility’s SOPs and sanitary and epidemiological rules SR 2.2.1.3218-14 “Sanitary and Epidemiological Requirements for the Device, Equipment, and Maintenance of Experimental Biological Clinics (Vivariums)” approved by the Resolution of the Chief State Sanitary Physician of the Russian Federation of 29 August 2014, No. 51. 

The animals were kept in the autoclaved sterile polycarbonate cages with Lignocel Wood Fibers wood pellets (JRC, Rosenberg, Germany) as bedding and fed ad libitum using standard food and purified water, under the following conditions: 20–24 °C, relative humidity of 45–65%, and light/dark cycle of approximately 12 h per day. Housing conditions and experimental procedures were done with the approval and under the guidelines of the ethics committee of animal experimentation of Sechenov University (approval number 047).

### 2.2. “Betuvax-CoV-2” Preparation

A recombinant RBD/S21/S14-FcIgG1 (RBD-Fc) antigen of the SARS-CoV-2 comprises a C-terminal RBD sequence containing two additional S21P5 and S14P5 epitopes, fused with the Fc-IgG1. The S14P5 epitope is located in the SD1 subdomain. Apparently, the antibodies against this antigen region prevent the virus interaction with the ACE2-receptor. The S21P2 epitope is located in the S2 region and overlaps with the fusion peptide. Antibodies against this epitope may counteract the fusion of the virus with the cell. Additionally, the order of epitopes was changed to increase the stability of the protein. The antigen was mixed with a previously prepared adjuvant as described in [15] so that the final concentration of RBD antigen was 40 μg/mL or 10 μg/mL, and the adjuvant was 400 μg/mL. The components were thoroughly mixed and placed in a shaker in the refrigerator for 3 h. The vials with the vaccine were then stored in a refrigerator at 2–8 °C.

### 2.3. Safety Studies

#### 2.3.1. Acute Toxicity Study

An acute toxicity study has been performed on the outbred mice (*n* = 120; 60 M and 60 F) and the outbred rats (*n* = 120; 60 M and 60 F). The animals were divided into groups according to their body weight (the requirement was that the difference between the individual body weight and the mean is less than 10%). Both the mice and rats were administered the vaccine intravenously or intramuscularly once at a dose of 10 μg or 40 μg or placebo (*n* = 20 per group; 10 M and 10 F). The body weight of the animals was determined at the beginning of the experiment (as previously indicated), as well as on the 7th and the 14th day following the administration. Clinical signs of the animals were recorded daily. Animals were sacrificed on the 14th day of the experiment.

#### 2.3.2. Subchronic Toxicity Study

A subchronic toxicity study of the vaccine was carried out in the outbred rats (*n* = 60; 30 M and 30 F) and Chinchilla rabbits (*n* = 24; 12 M and 12 F). Both groups of animals were administered the vaccine intramuscularly at a dose of 5 or 20 μg or placebo (rats: *n* = 20 per group; 10 M and 10 F; rabbits: *n* = 8 per group; 4 M and 4 F). The administrations were performed once every 10 days over a 30-day period (4 injections in total). The overall condition and the behavior of the animals were monitored daily, as well as the body weight and the symptoms of intoxication. On the 31st and the 44th day after the first administration, general and biochemical blood analysis, coagulometry, urinalysis, and behavioral reactions were performed. The animals were sacrificed on the 31st and the 44th day of the experiment.

#### 2.3.3. Reproductive Toxicity in Female Rats

The reproductive toxicity of the vaccine was studied in female (*n* = 60) and male (*n* = 30) outbred rats. Female rats were administered the vaccine intramuscularly at a dose of 5 or 20 μg or placebo once a week over a 15-day period before mating with males (3 injections in total, during 3 estrous cycles). A 0.9% sodium chloride solution was used as a placebo. Following the administrations, females were placed together with intact males (2:1) for 10 days (2 estrous cycles) (*n* = 30 per group, 10 M and 20 F). The overall condition and the behavior of the animals were monitored daily. The body weight of female rats was determined before the start of the study, after the last administration of the vaccine, and the animals were sacrificed on the 20th day of the gestation period. The first possible day of pregnancy was determined by the presence of spermatozoa in vaginal swabs.

On the 20th day of pregnancy, half of the females (*n* = 30) were sacrificed for the examination of the reproductive system and pre- and post-implantation mortality (the number of corpus luteum in the ovaries and the number of implantation sites, live and dead fetuses in the uterus, fetuses mass, and their craniocaudal size). The fertility index was calculated as a ratio of the number of pregnant female rats to their total number. Half of the pregnant female rats from each group were monitored until the delivery of the offspring occurred. The offspring were then monitored for 35 days continually and evaluated on general physical condition, behavior, body weight change, and mortality. 

#### 2.3.4. Reproductive Toxicity in Male Rats

Reproductive toxicity of the vaccine was studied in female (*n* = 60) and male (*n* = 30) outbred rats. Male rats were administered the vaccine intramuscularly at a dose of 5 or 20 μg or placebo once a week for 48 days before mating with females (7 injections in total). A 0.9% sodium chloride solution was used as a placebo. At the end of the experiment, intact female rats were placed together with male rats for mating (1:2) for 10 days (*n* = 30 per group, 10 M and 20 F). On the 20th day of the gestation period, half of the intact female rats and all male rats were sacrificed for the examination of the reproductive system (histological and morphological analysis of the testicles, functional state of the spermatozoa, and the index of spermatogenesis, in addition to the same analyses that were used in the previously described experiment). Half of the pregnant female rats were monitored until the offspring were delivered. The offspring were continually monitored for 35 days for general physical condition, behavior, body weight change, and mortality.

#### 2.3.5. Embryo- and Fetotoxicity Study

Embryo- and fetotoxicity study of the vaccine was performed on mature female (*n* = 60) and male rats (*n* = 30). Sexually mature males were kept with females (1:2) for 2 estrous cycles (10 days) (*n* = 30 per group, 10 M and 20 F). The first possible day of pregnancy was determined by the presence of spermatozoa in vaginal swabs. Pregnant female rats were administered the vaccine intramuscularly at a dose of 5 or 20 μg or placebo once a week till the 19th day of the pregnancy (3 injections in total). Half of the pregnant females were monitored until the offspring were delivered and evaluated on general physical condition, behavior, and body weight changes. Three to four days prior to the delivery, each female rat was placed in a separate cage to record the childbirth, the duration of the gestation period, and lactation. 

The number of the pups, live and stillborn fetuses, and mortality were evaluated over a 35-day period post-delivery period. The body weight of the pups was determined at birth and on the 4th, 7th, 14th, 21st, 28th, and 35th day following the delivery. The craniocaudal size was measured on the 4th day. The appearance of the coat, opening of the eyes, detachment of the auricle, as well as motor activity, emotional reaction, and the ability to coordinate movements were continually monitored and recorded. The effect on the rate of maturation of sensory-motor reflexes during the feeding period was studied using the following tests: negative geotaxis, flipping on a plane, cliff avoidance, and flipping in free fall tests.

### 2.4. Immunogenicity

The immunogenicity was studied in mice, golden hamsters, and Chinchilla rabbits. The C57BL/6 mice (*n* = 22) were immunized intraperitoneally, twice, at a dose of 5 or 20 μg/animal (*n* = 7 per group) or PBS (*n* = 8) over a 21-day period. The blood sera were taken on the 21st and the 43rd day of the experiment.

The BALB/c mice (*n* = 8) were administered the vaccine intraperitoneally twice at a dose of 5 μg/mouse (*n* = 5) or PBS (*n* = 3) over a 10-day period. The blood sera were taken on the 10th and the 24th day of the experiment.

Outbred mice (*n* = 20 per group) and Chinchilla rabbits (*n* = 8 per group) were administered two intramuscular shots at a dose of 5 or 20 μg or placebo (0.9% NaCl) over a 21-day period. Blood serum samples were taken from the rabbits on the 21st and the 35th day and from the mice on the 21st and the 42nd. 

Hamsters (*n* = 36) were administered the vaccine intramuscularly at a dose of 5 or 20 µg/animal or placebo (0.9% NaCl) (*n* = 12 per group, 6 M and 6 F) twice, over a 28-day period (the 1st and the 29th day of the experiment). On the 36th day of the experiment, the blood samples from the hamsters were collected for the neutralizing antibody titers analysis.

### 2.5. Anti-SARS-CoV-2 S-Protein Specific IgG by ELISA

Total immunoglobulins against the SARS-CoV-2 were determined using the “SARS-CoV-2-CoronaPass test system” (Biopalitra, Moscow, Russia). The antibody titers were expressed as log base 2 (Log_2_). The conversion to the logarithmic titers is presented in Table 1.

Neutralizing antibodies against the SARS-CoV-2 in hamster blood plasma were determined using the “SARS-CoV-2 Surrogate Virus Neutralization test kit” (GenScript, Piscataway, NJ, USA). 

Enzyme immunoassays were carried out using the “Multiskan GO” microplate spectrophotometer (Thermo Fisher Scientific, Waltham, MA, USA) at 450 nm.

### 2.6. Protective Efficacy

The protective efficacy of the vaccine was evaluated in a study using hamsters (*n* = 72) and rhesus monkeys (*Macaca mulatta*) (*n* = 18). Hamsters were administered the vaccine intramuscularly at a dose of 5 or 20 μg/animal (0.5 mL/animal) or intranasally at a dose of 4 μg (100 µL/animal, 10 µL in each nostril 5 times a day with an interval of 30 min). The control group received a placebo (0.9% NaCl) intramuscularly or intranasally or was not administered any solution (virus dose control group) (*n* = 12 per group, 6 M and 6 F). Healthy male rhesus monkeys (*n* = 18) received 0.5 mL of the vaccine intramuscularly at a dose of 5 or 20 μg/animal or placebo (0.9% NaCl) (*n* = 6 per group). The vaccine was administered twice on the 1st and the 29th day of the experiment (over a 28-day period). Following the immunization, the animals were infected intranasally with the SARS-CoV-2 at a dose of 10^5^ PFU (PFU—plaque-forming unit).

#### 2.6.1. Infection of Animals with the SARS-CoV-2

For the preparation of the infectious mixture, a SARS-CoV-2 virus isolate B (WA/1/2020) culture was used in the form of a freeze-dried 30% Vero C1008 cell suspension in a 10% sucrose water solution with the addition of a 10% fresh chicken egg yolk. The initial biological activity of the culture was 7 ± 0.2 lg PFU/mL. Content of two ampoules containing the cell culture was resuspended in Hank’s solution supplemented with 2% FCS (fetal calf serum) (Thermo Fisher Scientific, Waltham, MA, USA). The solution was administered intranasally at a dose of 10^5^ PFU/animal.

For further experiments, lung samples were taken from 4 hamsters and 2 monkeys from each group on days 2, 4, and 6 following the infection. The SARS-CoV-2 accumulation titer in the lungs was determined using the plaque assay on the Vero C1008 cell culture. Lung samples from macaques were used for histology and immunohistology analysis. 

#### 2.6.2. Determination of the VSI (Virus Suppression Index) of the SARS-CoV-2 in the Lungs of Golden Hamsters

Hamster lungs were homogenized and diluted in Hank’s solution with the addition of 2% FCS and antibiotics (streptomycin sulfate and benzylpenicillin sodium salt, 200 U/mL) (Thermo Fisher Scientific, Waltham, MA, USA) to prepare a 10% lung suspension. The biological activity of the virus from the lung samples was determined by titrating the prepared suspension to a monolayer of Vero C1008 cell culture in plastic vials with a 25 cm^2^ working surface area (CellStar, Greiner Bio-One GmbH, Kremsmünster, Austria). After removing the medium, a monolayer of Vero C1008 cells was infected with 10-fold dilutions of the virus. Vials with an infected monolayer were incubated for 1 h at 36.5–37.5 °C. The inoculum was decanted and the primary agar coating, prepared according to the prescription for the SARS-CoV-2, was added. The cell monolayer was incubated for 2 days at 36.5–37.5 °C, after which a secondary agar coating was added and incubated for 24 h at the same temperature. After the completion of this step, the number of negative colonies was determined. A decrease in the level of viral load at the peak of infection was taken as a parameter for evaluating the vaccine efficacy (at a 95% confidence level—decrease level from 1.2 to 1.8 lg, at 99% confidence level—more than 1.8 lg).

#### 2.6.3. Histological Study

In this study, 18 lung complexes from monkeys were examined, including trachea, main bronchi, left and right lungs, esophagus, fragments of the aorta, fat tissue of the mediastinum, thymus, and lymph nodes (the sections were fixed in a 10% formalin). The material was excised with macroscopic photo documentation. As a result, over 120 diagnostically significant areas of the lungs were obtained for histopathological micropreparations.

Using a computer video system (IBM PC + microscope Leica DM 1000, Leica Camera, Wetzlar, Germany) and the ImageScope-M software package (Systems for Microscopy and Analysis, Moscow, Russia), the “airiness” parameter, specific area of the histological section occupied by air and free from infiltration or exudate from the alveolar space, was determined in a semi-automatic mode.

An immunohistochemical study was performed to immunotype infiltrated cells in the lungs using antibodies against the T-lymphocyte marker (CD3—Cell Marque, 103-R94, Rocklin, CA, USA) and macrophages M2-phenotype (CD163 MsmAb—Cell Marque, 163M-15, Rocklin, CA, USA). Antibodies against vascular endothelial cells (CD31 Mon. mouse—Cell Marque, JC-70, Rocklin, CA, USA) were also used. The morphometric evaluation consisted in counting the number of cells per 1 mm^2^ area of the lung parenchyma.

For immunohistochemical evaluation of the coronavirus proteins (S- and N-proteins), SARS-CoV2 spike (Genetex, GTX135356, Irvine, CA, USA) and SARS-CoV2 nucleocapsid (Invitrogen, MA1-7404, Waltham, MA, USA) antibodies were used. The result was evaluated visually and qualitatively. The lung tissue from a patient who died from a coronavirus infection served as a positive control. The negative control was the lung tissue from a patient who died from causes unrelated to the respiratory system.

### 2.7. Statistical Analysis

The obtained data were analyzed with Microsoft Office Excel 2010 (Microsoft, Redmond, WA, USA) and GraphPad Prism v6.01 software (GraphPad Software, San Diego, CA, USA) for geometric mean, standard deviation, arithmetic mean, and standard errors. No samples or animals were excluded from the analysis. The Kruskal–Wallis H test, or its parametric equivalent one-way analysis of variance (ANOVA), was used for the analysis of variance between the group means, with subsequent pairwise comparison using the Tukey test or nonparametric pairwise multiple comparisons by Dunnett’s test. Values *p* < 0.05 were accepted as statistically significant.

## 3. Results

### 3.1. “ Betuvax-CoV-2” Vaccine Safety in Toxicity Studies

#### 3.1.1. Acute and Subchronic Toxicity Studies

**Figure 1:**
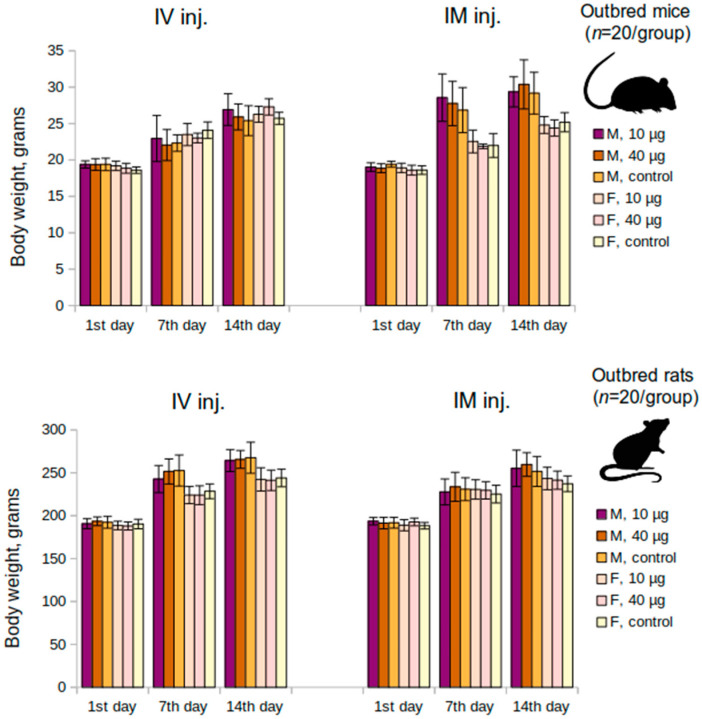
Mean changes of body weight over time in the acute toxicity study in mice and rats groups. M—males, F—females, IM inj.—intramuscular injection, IV inj.—intravenous injection; *p* > 0.05.

The acute toxicity study in outbred mice and rats with single intravenous and intramuscular vaccine injections demonstrated that a single administration of the vaccine at 10 μg/animal or 40 μg/animal did not lead to mortality, body, or organ weight decrease, or worsening of the overall condition. The determination of lethal doses LD10, LD16, LD50, or LD84 was impossible due to the absence of mortality. It was also shown that the animals steadily gained weight with no lags for the entire period of the study. The average values of the initial body weight and the body weight at the end of the study are depicted in Figure 1. A comparison of six groups (males and females separately) using the Kruskal-Wallis test did not show any significant differences (*p* > 0.05). The necropsy study, carried out on the 14th day following the vaccine administration, showed no abnormalities in the structure of the internal organs in mice and rats or any pathological changes (data not shown).

Subchronic toxicity studies in rats and Chinchilla rabbits showed that there were no differences observed between the experimental group and the control in terms of the activity, movement, appearance, physiological functions, animal behavior, bone marrow cellularity or myelogram parameters, body weight, based on hematological, biochemical, or coagulometric parameters and urine analysis.

The necropsy study did not reveal any deviations in the structure of the body and the internal organs in experimental groups. There were also no intergroup differences in the mass of internal organs (heart, lungs, liver, kidneys, brain), including immunocompetent organs (thymus, spleen). Histological examination of the internal organs and tissues in experimental animals and the control demonstrated no pathological changes (data not shown).

The absence of toxicity, including the lack of immunotoxicity and local irritation, was demonstrated for the “Betuvax-CoV-2” vaccine in the acute and subchronic toxicity studies in mice, rats, and rabbits.

#### 3.1.2. Reproductive Toxicity Study in Female Rats

**Figure 2:**
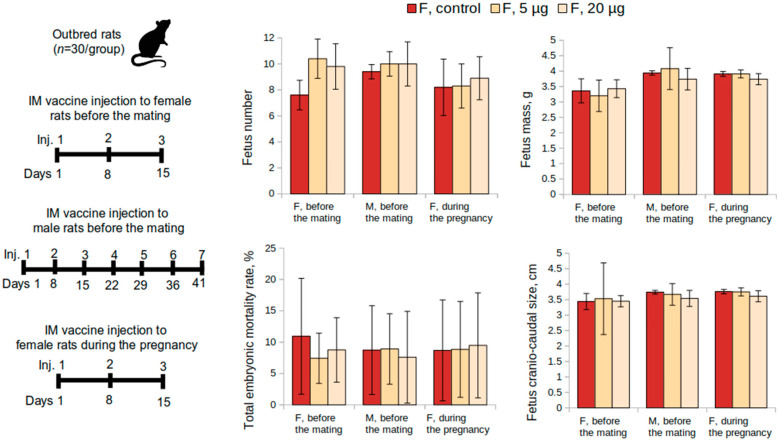
Examination of the reproductive system according to the following parameters: fetus number, fetus mass, total embryo mortality rate, fetus craniocaudal size from female rats intramuscularly immunized with the “Betuvax-CoV-2” vaccine 15 days before mating (F, before the mating), before mating with males previously immunized during a 48-day period (M, before the mating), and during the first 19 days of the pregnancy (F, during the pregnancy). F—females, M—males; IM—intramuscular administration; *p* > 0.05.

To evaluate the condition of the reproductive system of pregnant female rats, immunization was performed 15 days before mating. The administration of the “Betuvax-CoV-2” vaccine at doses of 5 and 20 µg/animal did not affect the reproductive function adversely (Figure 2). The embryo mortality at all stages of the development in female rats immunized with the vaccine did not differ from the control. There were also no significant differences in fetus weight or size (*p* > 0.05, Kruskal-Wallis test). 

Macroscopic and histological examination of rats’ ovaries that were immunized with the “Betuvax-CoV-2” vaccine within 15 days before mating showed no signs of pathological changes. The ovaries in all groups were dark red, moderately dense, and with an uneven surface. Follicles of various sizes and degrees of maturation were visible. The follicular epithelium was not changed; the nuclei were light and clear, and the ovarian medulla was full-blooded (data not shown).

**Figure 3:**
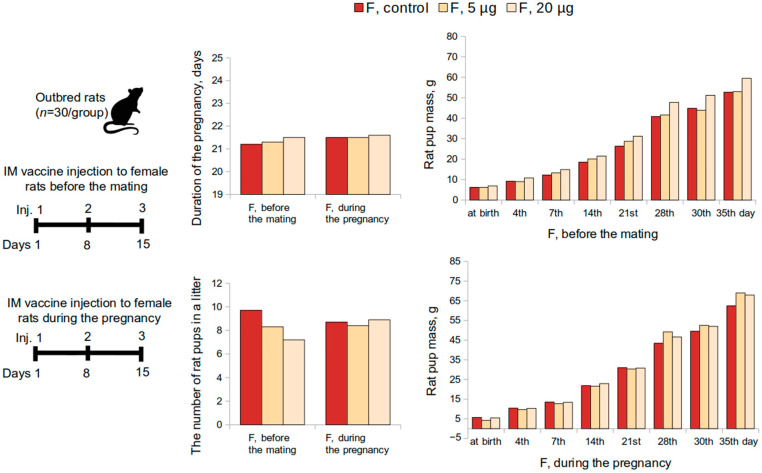
Evaluation of postnatal development of the offspring according to the following parameters: duration of the pregnancy, the number of rat pups in a litter, rat pup mass, when female rats were intramuscularly immunized with the “Betuvax-CoV-2” vaccine 15 days before mating (F, before the mating); during the first 19 days of the pregnancy (F, during the pregnancy). F—females; IM—intramuscular injection; *p* > 0.05.

The effect of the “Betuvax-CoV-2” vaccine, when administered to female rats 15 days before mating, was assessed on the postnatal development of the offspring (Figure 3). Half of the pregnant rats were monitored continuously until the delivery of the offspring. The development of the offspring was observed for 35 days for general physical condition, behavior, weight dynamics, and mortality. All pregnant animals in the experimental groups demonstrated normal birth and labor processes. The general and specific indicators of postnatal development did not differ significantly from the control values (*p* > 0.05, Kruskal-Wallis test). The duration of the pregnancy in experimental females was in accordance with the physiological norm for this type of animal and the control. All rat pups were born viable and no mortality during the first month was recorded. The physical development of the offspring proceeded without any deviations.

#### 3.1.3. Reproductive Toxicity Study in Male Rats

The condition of the reproductive system of the pregnant rats who were mated with the males who were previously administered the “Betuvax-CoV-2” vaccine for 48 days before mating (at doses of 5 μg and 20 μg) is depicted in Figure 2. The values indicating the condition of the reproductive system did not differ from those females mated with the males of the control group (*p* > 0.05, Kruskal–Wallis test). In this way, the administration of the vaccine did not adversely affect the reproductive function of male rats.

A macroscopic testicle study in outbred male rats that were intramuscularly administered the “Betuvax-CoV-2” vaccine at doses of 5 μg and 20 μg once a week for 48 days demonstrated no pathological changes. Testicles of all experimental groups were unchanged compared to the control, oval, pale gray in color; the blood vessels with well-expressed lobulated structure (data not shown). 

Histological study in rat testicles showed that the administration of the vaccine at 20 μg once a week for 48 days had an adverse effect on the condition of the spermatogenic epithelium expressed in abnormal polarity and germ cell localization. It manifests as cell desquamation into the lumen of seminiferous tubules (in 6 out of 10 males of the 20 μg group). The spermatogenesis index of the rats administered the vaccine at 5 μg did not show significant intergroup differences (*p* > 0.05, Dunnett’s test). However, the 20 µg group was significantly different from the control (*p* < 0.001, Dunnett’s test), the absolute difference between the sample means in the control group, and the 20 µg group was only 0.22. Thus, the spermatogenesis in the 20 μg group decreased slightly and had no pronounced effect on the reproductive potential of male rats.

Statistically significant intergroup differences in the functional state of the spermatozoa of rats that were administered the “Betuvax-CoV-2” vaccine at 5 and 20 μg once a week for 48 days were also not detected. There were no adverse effects on the embryo development recorded. This was confirmed by examining the reproductive system of pregnant females and the development of the offspring (Figure 2).

#### 3.1.4. Embryo- and Fetotoxicity Study

Embryo- and fetotoxic effects of the vaccine were examined during the antenatal period in cases when the vaccine was administered intramuscularly to female rats during the first 19 days of the pregnancy. Intramuscular injections were administered once a week from the 1st to the 19th day of pregnancy at 5 or 20 μg and did not show embryotoxic and teratogenic effects in terms of the offspring development. Mortality was absent and there were no signs of toxicosis, visible pregnancy abnormalities, or decreased body weight gain of female rats in the experimental group in comparison to the control. Embryo mortality at all stages of the development at all doses did not differ from the corresponding values of the control group (0%). Significant differences from the control in weight and size of the fetus and placenta were also not revealed (Figure 2). No external abnormalities of the embryos were found. The study of nine sagittal sections confirmed the absence of internal developmental anomalies. No anomalies in the skeleton development and delays in the ossification of the skeleton of the embryos were also found (data not shown).

The effects of intramuscular administration of the vaccine on female rats during the first 19 days of pregnancy were evaluated during the postnatal development of the offspring as well (Figure 3). Integral and specific indicators of postnatal development of rat pups from experimental female rats did not differ significantly from the control values (*p* > 0.05, Kruskal–Wallis test). None of the doses affected the physical development of the offspring, the development of sensory-motor reflexes during the period of feeding, motor activity, or emotional reactions after the end of feeding.

The physical development of the offspring was normal. The recorded time of the manifestation of specific developmental events, such as the detachment of the auricles, the appearance of the primary hairline, or the opening of the eyes, were within the control and standard fluctuations for this animal species. Embryos with developmental anomalies, both external and internal, were absent from all experimental groups.

Thus, the results of the preclinical study indicate a high safety, absence of general toxicity, or reproductive toxicity of the “Betuvax-CoV-2” vaccine in mice, rats, and Chinchilla rabbits of both sexes.

### 3.2. “Betuvax-CoV-2” Vaccine Stimulates Humoral Immune Response 

#### 3.2.1. Antibody Titers in the Sera of C57/Black and BALB/c Mice

**Figure 4:**
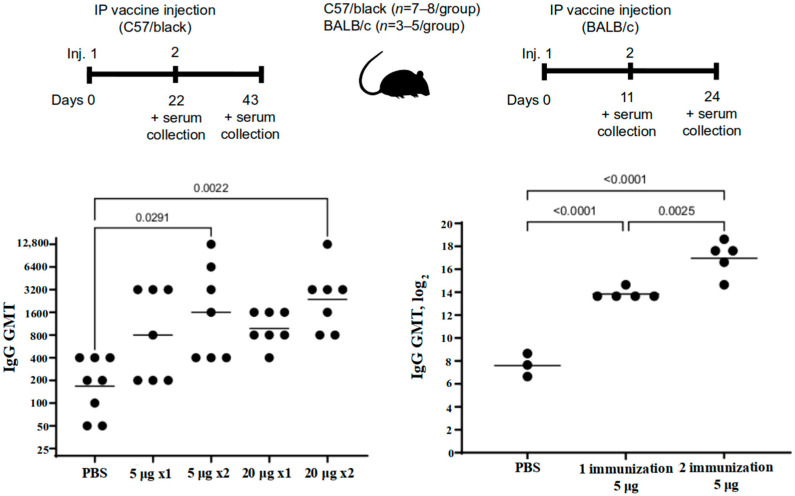
The level of antibodies against the SARS-CoV-2 RBD-based protein in the sera of immunized C57/black (**left**) and BALB/c (**right**) mice after a single (1×) or double (2×) intraperitoneal immunization with “Betuvax-CoV-2” at a dose of 5 μg or 20 μg (**left**) and 5 μg (**right**). Separate symbols (dots) represent individual values of titers in animals; the horizontal line shows average group geometric mean titers; *p* < 0.05.

Immunization and sample acquisition for this study were performed as shown in Figure 4, top panel. The study showed that the vaccine antigen-specific antibody level has increased after a single immunization with “Betuvax-CoV-2”, yet it was not considerable. A significant increase in specific antibody titers in the immunized C57/black mice in comparison with control was observed after two intraperitoneal injections of the vaccine preparation. The IgG titers significantly increased in both groups: upon administration of the vaccine at a dose of 5 µg/animal (GMT (geometric mean titer) = 1600) and at a dose of 20 µg/animal (GMT = 2377), compared to control (*p* < 0.05, Tukey’s test) (Figure 4).

A significant increase in specific antibody titers in the serum of BALB/c mice was registered after a single immunization compared to the control group (PBS). The GMT value after a single immunization at a dose of just 5 μg was 14,703. The antibody titers after re-vaccination compared with those after a single immunization were multiplied by 9 times (GMT = 132,578). All groups were significantly different from each other (*p* < 0.05, Tukey’s test) (Figure 4).

#### 3.2.2. Antibody Titers in the Sera of Rabbits and Outbred Mice

**Figure 5:**
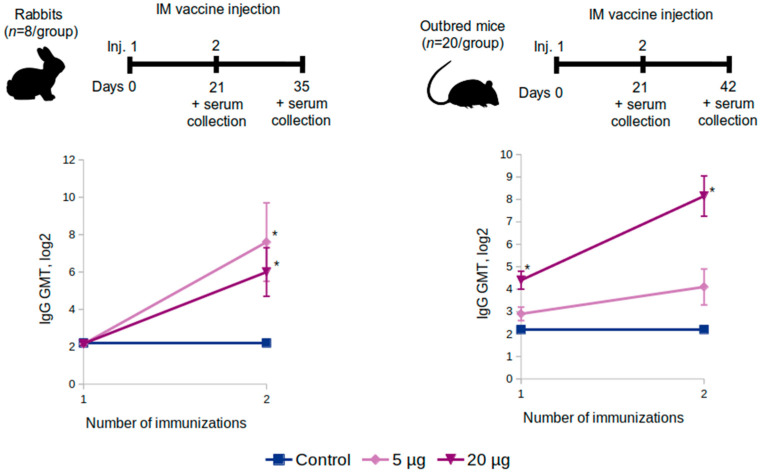
The growth of the GMT level of antibodies specific to the SARS-CoV-2 RBD protein in the serum of the intramuscularly immunized rabbits (**left**) and outbred mice (**right**) with “Betuvax-CoV-2” at 5 and 20 µg/animal; * *p* < 0.05.

The study in rabbits and mice was performed according to the scheme in Figure 5. The study on rabbits showed that the RBD-specific antibody level after the first immunization in the 5 μg, 20 μg, and the control groups did not differ significantly (*p* = 0.999, Kruskal–Wallis test), yet in the outbred mice, it significantly differed (*p* < 0.001, Kruskal–Wallis test) in the 20 µg group (Log_2_ = 4.40, GMT = 1:21) (*p* < 0.001, Dunnett’s test).

In rabbits, 14 days after the second immunization (on the 35th day of the experiment), the antibody level in the 5 μg, 20 μg, and the control groups differed significantly (*p* = 0.044, Kruskal–Wallis test). The level of antibodies increased in the 5 μg group (Log_2_ = 7.57, GMT = 1:190) (*p* = 0.024, Dunnett’s test) as well as in the 20 μg group (Log_2_ = 6.07, GMT = 1:67) (*p* = 0.04, Dunnett’s test) compared with the control (Figure 5).

In the outbred mice, 21 days after the second immunization, on the 42nd day after the first administration of the vaccine, the GMT of antibodies in the 5 µg, 20 µg, and control groups was significantly different (*p* < 0.001, Kruskal–Wallis test). In the 5 μg group, the level of antibodies did not significantly increase (Log_2_ = 3.99, GMT = 1:16) (*p* = 0.088, Dunnett’s test) compared to the control group. In the 20 μg group, the antibody level significantly increased (Log_2_ = 8.24, GMT = 1:305) (*p* < 0.001, Dunnett’s test) compared to the control (Figure 5).

#### 3.2.3. The Level of Neutralizing Antibodies in the Sera of Golden Hamsters

The presence of neutralizing antibodies against the SARS-CoV-2 was determined by the “SARS-CoV-2 Surrogate Virus Neutralization Test Kit” (GenScript, Piscataway, NJ, USA) in the blood plasma of hamsters taken on the 36th day after two immunizations. The first group of hamsters with an intramuscular placebo injection (0.9% NaCl) showed the absence of antibodies against the SARS-CoV-2. The second group of hamsters, immunized intramuscularly with “Betuvax-CoV-2” at 5 µg/animal, showed a response in the production of neutralizing antibodies in 33.3% of the animals (2 out of 6 females and 2 out of 6 males). The third group of hamsters immunized intramuscularly at 20 μg/animal produced neutralizing antibodies in 92% of cases (5 out of 6 males and 6 out of 6 females) (Table 2).

To conclude on the immunogenic studies shown above, “Betuvax-CoV-2” demonstrated that all animal species (mice, rats, rabbits, hamsters) produced virus-neutralizing antibodies against the RBD component of the SARS-CoV-2.

### 3.3. “Betuvax-CoV-2” Vaccine Shows High Protective Efficacy against the SARS-CoV-2 

#### 3.3.1. Accumulation of the SARS-CoV-2 Virus in the Lungs of Golden Hamsters

**Figure 6:**
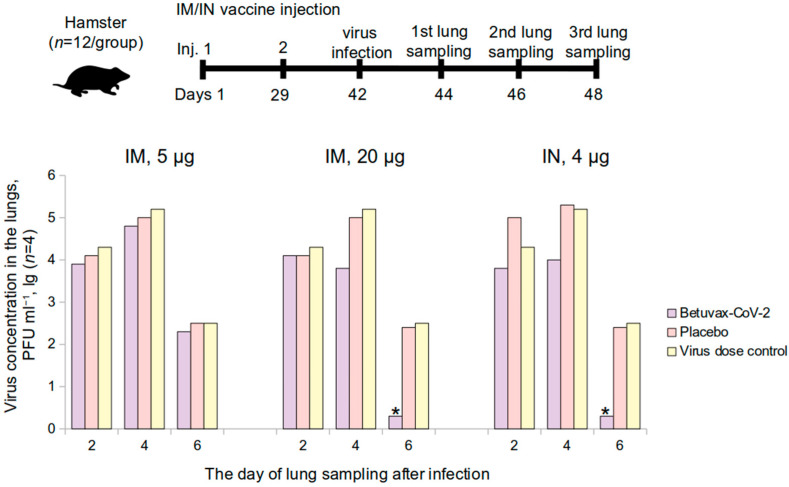
The level of the SARS-CoV-2 accumulation in the lungs of vaccinated golden hamsters on the 2nd, 4th, and the 6th day after infection. IM—intramuscular, IN—intranasal administration; * *p* < 0.05.

The specific antiviral activity of the vaccine (protective efficacy) was evaluated in golden hamsters at 5 μg and 20 μg (intramuscularly) and 4 μg (intranasally) before the infection with SARS-CoV-2. The experiment was performed on hamsters as described in Figure 6, top panel. Neither in males nor females were there clinical signs of deviations of health condition, local irritation, or statistically significant intergroup differences in terms of body weight or body temperature during the immunization period (data not shown).

After an intranasal infection with the SARS-CoV-2 at 10^5^ PFU in the 20 μg/animal hamster group (double intramuscular injection) and in the 4 μg/animal group (double intranasal injection), the virus concentration in the lungs was completely suppressed on the 6th day of the study in comparison with placebo (Figure 6).

The Virus Suppression Index (VSI) in the hamster lungs is presented in Table 3. In the 20 μg/animal hamster group, the VSI was: 1.4 ± 0.2 lg PFU·mL^−1^—on the 4th day after the infection, 2.2 ± 0.2 lg PFU·mL^−1^—on the 6th day after infection. In the group that was immunized intranasally at a dose of 4 µg/animal (100 µL of a dosage of 40 µg/mL): 1.3 ± 0.2 lg PFU·mL^−1^ and 2.2 ± 0.2 lg PFU·mL^−1^ on the 4th and 6th day, respectively. When the hamsters were immunized intramuscularly at a dose of 5 µg/animal, the VSI ranged from 0.2 ± 0.3 to 0.4 ± 0.4 lg PFU·mL^−1^. In accordance with the Guidelines for Preclinical Studies [16], the vaccine is effective if the VSI is from 1.2 to 1.8 lg with *p* < 0.05, at 1.8 lg or more with *p* < 0.01. 

Therefore, in the groups that were vaccinated intramuscularly (20 µg/animal) and intranasally (4 µg/animal), the VSI on the 4th day was in the range from 1.2 to 1.8 lg indicating the vaccine efficacy with *p* < 0.05. The VSI was above 1.8 lg on the 6th day in the intramuscular 20 μg administration and intranasal 4 μg administration groups, which testifies a complete absence of the virus production in the lungs, indicating vaccine protectiveness with *p* < 0.01.

Thus, it has been shown that the protectiveness of a double intramuscular administration of the vaccine at 20 μg and the intranasal administration at 4 μg against the SARS-CoV-2 was 99%.

As the level of the SARS-CoV-2 accumulation in the lungs of the immunized hamsters was lower than in the control group, it might also suggest that there was no ADE (antibody-dependent enhancement) effect in the short-term period of observation.

#### 3.3.2. Evaluation of the Protective Activity of the “Betuvax-CoV-2” Vaccine against the SARS-CoV-2 in the Lungs of Rhesus Monkeys

**Figure 7:**
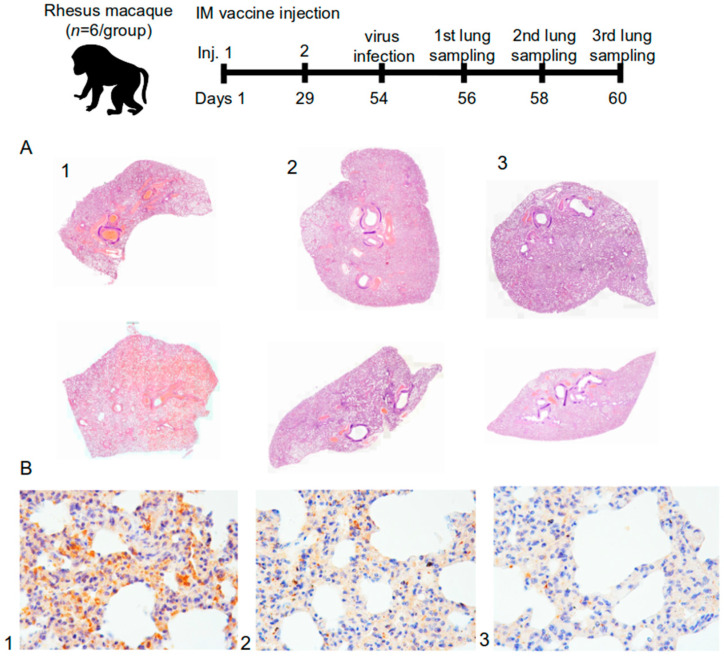
(**A**) Histotopogram of macaque representative lung samples taken on the 6th day after the infection of rhesus macaques with the SARS-CoV-2; (**B**) IHC-reaction to S-protein (brown reaction product) in representative lung tissues of rhesus macaque (1—control, 2—5 μg dose, 3—20 μg dose); stained with hematoxylin; magnification 250× (*n* = 72).

The protective activity of the vaccine was studied in intramuscularly immunized rhesus macaques at 5 or 20 μg before the infection with the SARS-CoV-2 (Figure 7, top panel). There were no clinical signs of changes in health condition, local irritation, or significant intergroup differences in body weight or body temperature during the immunization period (data not shown).

After the macaques were intranasally infected with the SARS-CoV-2 at 10^5^ PFU, there were changes in pathohistology, such as interstitial inflammation and widespread (in pulmonary circulation) thrombosis (thrombovasculitis) of large, medium, and small vessels in the lung tissues. However, the tissue changes in the animals of the control group (1) were significantly different from those in the vaccine groups of 5 µg and 20 µg (2 and 3). In the control group, the pathomorphological picture showed signs of exudative inflammation and catarrhal changes in the bronchi. The most characteristic symptom was thrombo-hemorrhagic changes. In the vaccine groups, there was a slight hemorrhagic component, however, with a more pronounced manifestation of interstitial inflammation in the form of lympho-macrophage infiltration. Also, in these groups, there were no progressive lesions, necrosis, abscess formation, or signs of fibrinous effusion in the alveoli (Figure 7A).

The airiness index expresses the specific area in the lumen of alveoli on a histological section participating in external respiration and gas exchange. According to the airiness index, the number of differences found between the groups, such as signs of damage, were probably of viral origin. In fact, the presence of normal airiness foci was registered only in the lungs of animals of the vaccine groups (Figure 7A).

Tracheal injury included less pronounced signs of partial desquamation of the epithelium at the level of the lower third, as well as a minimally pronounced parietal accumulation of mucofibrinous exudate (in the control group). The animals in the vaccine groups observed no changes in the trachea for the duration of the study.

#### 3.3.3. Immunohistochemical and Morphometric Characteristics of the Inflammatory Infiltrate

Immunohistochemical evaluation of the tissue samples was carried out using anti-human antibodies against CD163, a marker of macrophages (M2, histiocytes), and antibodies against CD3, a panlymphocyte marker. Lung tissue from a deceased person confirmed to be infected with the novel coronavirus infection was used as a positive control. 

The lymphocyte-macrophage ratio was the highest in the control group on the 6th day—at about 70% (66.7%). Therefore, about two-thirds of the leukocytes in the infiltrate were T-lymphocytes. The most pronounced immune response at the level of tissue and according to the infiltrate characteristics was in the control group. More pronounced inflammatory manifestations were specific to the animals in the experimental groups, and the maximum number of macrophages was in the 20 μg group.

As it is shown in Figure 7A, lung samples from the control group (1, Figure 7A) were consistent with subtotal hemorrhagic interstitial pneumonia. Segmental bronchi in the distal sections were filled with blood, full of fibrin and many leukocytes. All vessels were sharply plethoric, mostly thrombosed. Subpleural vessels were especially pronounced with leukocyte infiltration. There were also some foci of effusion pleurisy. In Figure 7A, on the upper part of the control group (1, Figure 7A), it can be seen that the lobar bronchus was entirely filled with blood. Dystelectasis, thickening of the interalveolar septa due to edema and massive leukocyte infiltration, and subtotal thrombosis of the vessels of all calibers were recorded as well.

For the group with a dose of 5 μg (2, Figure 7A) in both samples, the lobar and segmental bronchi were passable; there was no secret or exudate. Focal interstitial edema, infiltration, and partial thrombosis of large and a part of small vessels were also observed. In both samples (2, Figure 7A), areas of high airiness were also determined.

The group immunized with 20 μg (3, Figure 7A) was characterized by passable lobar and segmental bronchi and the absence of exudate. Peribranchial focal lympho-macrophage infiltration and focal changes were absent. Some blood vessels were either thrombosed or contained sludge, but no hemorrhagic component was observed. On the upper part (3, Figure 7A), the stroma of the organ was diffusely infiltrated with leukocytes; there were no segmented leukocytes or focal changes. In these groups (3, Figure 7A), large areas of high airiness and single lymphoid nodules were also observed.

#### 3.3.4. Detection of Viral Proteins in Lung Tissue

The mosaic arrangement of lung tissue areas with virus S-protein was determined by immunohistochemistry (IHC) on the 2nd, 4th, and 6th day after infection. Reactions with antibodies against the N-protein turned out to be negative both with the experimental material and with the control samples. Figure 7B shows that there was a trend toward a decrease in the detection of the S-protein in the lungs of primates depending on the use of the “Betuvax-CoV-2” vaccine and its dosage. The detectable protein inclusions of the S-protein in groups 5 µg and 20 µg (2 and 3, respectively, Figure 7B) were significantly less compared to the control group (1, Figure 7B).

Moreover, the ADE effect was evaluated according to the criteria of overall morphological and physiological state, as well as macroscopic changes in the lungs of the vaccinated and the control animals. Macroscopic assessment of the lungs of monkeys, as well as the immunohistochemical analysis of the accumulation of the S-protein of the SARS-CoV-2 in the lungs of the vaccinated animals, showed no ADE effect, supporting our previous findings in hamsters.

As a result of determining the protective activity of the “Betuvax-CoV-2” vaccine in intramuscularly immunized hamsters at a dose of 20 μg/animal and intranasally at a dose of 4 μg/animal, an elimination of the virus from the lungs was shown to be 99%. Histology of rhesus monkey tissue also showed that animals vaccinated intramuscularly at 5 or 20 μg did not demonstrate pronounced signs of damage to the vascular component, unlike the control group, but reflected an inflammatory component of the immune system to a greater extent. Also, IHC identification of the S-protein showed a significantly lower presence in the vaccinated macaques. In the study of the protection activity in both hamsters and rhesus monkeys, no manifestations of the ADE effect were recorded during the observation. 

## 4. Discussion

The present preclinical study of the “Betuvax-CoV-2” vaccine was conducted to evaluate its safety and efficacy. The vaccine was studied in various toxicity studies (acute and subchronic toxicity) and, as a result, it was proved to be safe for use. Mortality was zero, the animals gained body weight, the mass of the internal organs did not change, and no pathologies were recorded. Also, there were no signs of immunotoxicity or local irritation.

Reproductive toxicity tests in female rats had shown that the administration of the vaccine at a dose of 5 or 20 μg, both before mating and during pregnancy, did not adversely affect the reproductive function or had teratogenic effects on the development of the offspring. The course of the pregnancy and the pup’s postnatal development did not differ from the control parameters. The mortality of the pups was zero.

Multiple administration of the vaccine (7 times within a 48-day period) to male rats as part of the reproductive toxicity study did not adversely affect the pregnancy of the females and the postnatal development of their offspring. Macroscopic examination of the testicles revealed no pathological changes. However, a histological study showed that male rats who received the vaccine at a dose of 20 μg 7 times during a 48-day period showed some change in the spermatogenic epithelium. Nevertheless, the spermatogenesis index in this group decreased insignificantly and did not affect the reproductive potential of the male rats. The functional state of spermatozoa did not differ from the control group as well. 

It should also be noted that the subchronic toxicity study revealed no similar changes in spermatogenic epithelium, as well as other signs of reproductive toxicity in males. The changes revealed in the male reproductive toxicity study were probably associated with a high dose of the vaccine. In the subchronic toxicity study, the drug vaccine was administered 4 times intramuscularly (1 time in 10 days) or 2 times intramuscularly (with an interval of 21 or 28 days), while in this experiment, the males received 7 intramuscular injections of the vaccine (once per week) at a dose of 20 μg, corresponding to the human dose.

The study of immunogenic properties of the “Betuvax-CoV-2” vaccine has proved its ability to stimulate both humoral and cellular immune responses. Previously we showed [15] that two-dose immunization of the mice leads to the formation of an adaptive T-cell immune response. We observed an increase in both CD4+ and CD8+ Tem Spike-specific cells after two administrations. Moreover, based on the type of the antigen formulation (betulin adjuvant, size, and shape of nanoparticles), the Th1-directed polarization of the cytokine response with the level of the CD4+ Tem (IL-2 and TNFα) was determined. A statistically significant increase was also shown for the multifunctional CD8+ T cells (IFNγ+ IL-2+ TNFα+). This cytokine pattern is thought to be beneficial because patients with severe COVID-19 are usually characterized by decreased IFNγ levels and a shift to a more pronounced Th2 profile [17].

The administration of the “Betuvax-CoV-2” vaccine to mice (C57BL/6, BALB/c, outbred), Chinchilla rabbits, hamsters, and primates at a dose of 5 or 20 μg resulted in the increase of the titers of the RBD-specific antibodies, specifically after the second shot. Antibodies against the RBD component of the vaccine were neutralizing, thereby providing a protective immune response against the SARS-CoV-2.

The protective activity of the vaccine was studied in golden hamsters and rhesus monkeys after being infected with the SARS-CoV-2. In a study on the suppression of the virus production in the lungs of golden hamsters, it was shown that the protection level of the “Betuvax-CoV-2”, when administered intramuscularly at a dose of 20 μg, as well as intranasally at a dose of 4 μg, was 99%. Histological examination of the lungs of macaques also confirmed this finding. In the group of intramuscular injection of the vaccine, there were no pronounced signs of damage to the microvessels with developing hemorrhagic manifestations and subtotal vessel thrombosis of the pulmonary circulation as in the control group. The immunized groups did not show signs of strong intra-alveolar exudation, formation of hyaline membranes, or pathological secretion in the lumen of the bronchi and bronchioles. The immunophenotypic characteristic of the infiltrate in the later stages indicated a predominant cellular immunity. In addition, immunohistochemical detection of the S-protein in the lungs of the infected primates was markedly lower in the 5 µg and the 20 µg intramuscular vaccine groups. It should be noted that in the study of the protection activity in hamsters and macaques, the ADE effect of the vaccine was not recorded.

Some of the advantages of the “Betuvax-CoV-2” vaccine over other available vaccines are its high safety profile and comfort of use. As the “Betuvax-CoV-2” vaccine is a subunit protein vaccine, it does not contain any viruses such as Ad26.COV2.S (Janssen), Sputnik V (Gamaleya National Center of Epidemiology and Microbiology), Vaxzevria (AstraZeneca/Oxford University), or Convidecia (CanSino) adenovirus-based vaccines. Therefore, this vaccine does not deliver any genetic material, which could lead to unwanted inflammation and side effects, as previously described [13,18,19,20,21,22]. However, a rare, dangerous side effect of these types of vaccines is the vaccine-induced thrombotic thrombocytopenia (VITT) which was suggested to be associated with the use of viral vector components that bind to platelet factor 4 and form immunogenic complexes which attacked by the circulating IgG. Consequently, PF4/IgG complexes can bind to the surface of platelets and activate them, resulting in a prothrombotic state [23,24,25], although more studies are required to confirm the exact underlying mechanism. Moreover, previously generated immunity to the adenovirus capsid might prevent future uses of a booster vaccine, other adenoviral based-vaccines, or therapeutic agents. As the use of booster vaccines becomes more and more frequent with the rise of novel SARS-CoV-2 strains [26], it is important to note the possibility of using the “Betuvax-CoV-2” repeatedly in a single individual. 

The mRNA-based vaccines such as mRNA-1273/Spikevax (Moderna) or BNT162b2/Comirnaty (Pfizer/Biontech) are reported to be highly effective [27,28]. However, a great number of various side effects were reported as well [13]. This is in line with the fact that this type of vaccine is new with unknown long-term side effects. On the contrary, the “Betuvax-CoV-2” vaccine represents a more traditional vaccine development approach using a recombinant protein as an antigen. Furthermore, an increased risk of anaphylactic shock has been reported for Moderna and Pfizer vaccines [29] which could be associated with the adjuvant, the antigen, or the overall formulation. Meanwhile, the “Betuvax-CoV-2” has not been reported to cause any such effect. Additionally, mRNA-based vaccines often require extreme temperature conditions for long-term storage [30]. The “Betuvax-CoV-2”, on the contrary, can be stored at 2–8 °C in refrigerators for up to a year. This feature could be particularly attractive for countries that do not have an opportunity to keep the vaccine under such conditions.

Based on the design and the vaccine type, the closest vaccine to the “Betuvax-CoV-2” is Nuvaxovid/NVX-CoV2373 by Novavax. This vaccine has been shown to be efficient and safe [31], although it uses a different antigen based on the full-length Spike protein. Therefore, one more possible advantage of the “Betuvax-CoV-2” vaccine might arise from the antigen used rather than from the adjuvant platform. The “Betuvax-CoV-2” is based on the RBD of Spike protein, as opposed to the full-length Spike encoded in all registered vaccines. This could be an advantageous feature since the immune response elicited in response to full-length Spike could be broad in terms of the antibody repertoire, whereas only neutralizing antibodies against RBD would prevent the virus from entering the cells [32]. Moreover, the antibodies that are not specific to the RBD domain might then be involved in ADE [33,34], so the use of the RBD-based protein could be advantageous in terms of both safety and the efficacy of the vaccine. 

## 5. Conclusions

Thus, the results of the preclinical studies showed the immunogenicity (humoral and cell immunity), protection (significant reduction in virus loads), and safety of the vaccine, including the absence of the ADE effect, thus demonstrating a favorable “risk-benefit” ratio.

Given that there is active development and spread of new strains of the SARS-CoV-2 such as Omicron, there is still a high demand for frequent boosting and the use of polyvalent vaccines for different strains, which differ greatly in antigenic determinants. The “Betuvax-CoV-2” vaccine, due to its high safety profile, efficacy, and the possibility of scalable production for different emerging strains, is well suited as a booster vaccine. We are also considering the development of an intranasal vaccine against the coronavirus, which showed remarkable protection in this study. Currently, intramuscular injection of the “Betuvax-CoV-2” vaccine is currently being evaluated in a phase 1/2 of a randomized, double-blind placebo-control clinical trial (NCT05270954).

## Figures and Tables

**Table 1:** Logarithmic titer data in reverse titer.

	Conversion of Reverse Titer to Log_2_								
Reverse titer	1:5	1:10	1:20	1:40	1:80	1:160	1:320	1:640	1:1280
Log_2_	2.32	3.32	4.32	5.32	6.32	7.32	8.32	9.32	10.32

**Table 2:** Golden hamsters with neutralizing antibodies against SARS-CoV-2.

Hamsters with Neutralizing Antibodies	Control 0.9% NaCl *n* = 12 (6M, 6F)	Betuvax-CoV-2 (5 µg/Animal) *n* = 12 (6 M, 6 F)	Betuvax-CoV-2 (20 µg/Animal) *n* = 12 (6 M, 6 F)
Quantity of hamsters with neutralizing antibodies/ all hamsters	0/12	4/12	11/12

**Table 3:** Virus Suppression Index for vaccinated hamsters.

Hamster Groups	Virus Suppression Index, lg PFU·mL^−1^		
	2nd Day	4th Day	6th Day
5 µg/animal, IM	0.4 ± 0.3	0.4 ± 0.4	0.2 ± 0.3
20 µg/animal, IM	0.2 ± 0.2	1.4 ± 0.2 *	2.2 ± 0.2 **
4 µg/animal, IN	0.5 ± 0.2	1.3 ± 0.2 *	2.2 ± 0.2 **

In accordance with the Guidelines for Preclinical Studies [16], the drug is effective if the * VSI is from 1.2 to 1.8 lg (*p* < 0.05) * and if the VSI is at 1.8 lg or more (*p* < 0.01) **. IM—intramuscular, IN—intranasal administration.

## Data Availability

The datasets generated or analyzed during this study are available from the corresponding author on reasonable request.
